# Scalable network emulation on analog neuromorphic hardware

**DOI:** 10.3389/fnins.2024.1523331

**Published:** 2025-02-05

**Authors:** Elias Arnold, Philipp Spilger, Jan V. Straub, Eric Müller, Dominik Dold, Gabriele Meoni, Johannes Schemmel

**Affiliations:** ^1^European Institute for Neuromorphic Computing, Kirchhoff Institute for Physics, Heidelberg University, Heidelberg, Germany; ^2^Advanced Concepts Team, European Space Research and Technology Centre, European Space Agency, Noordwijk, Netherlands; ^3^Faculty of Aerospace Engineering, Delft University of Technology, Delft, Netherlands

**Keywords:** modeling, neuromorphic, spiking neural networks, virtualization, accelerator abstraction

## Abstract

We present a novel software feature for the BrainScaleS-2 accelerated neuromorphic platform that facilitates the partitioned emulation of large-scale spiking neural networks. This approach is well suited for deep spiking neural networks and allows for sequential model emulation on undersized neuromorphic resources if the largest recurrent subnetwork and the required neuron fan-in fit on the substrate. We demonstrate the training of two deep spiking neural network models—using the MNIST and EuroSAT datasets—that exceed the physical size constraints of a single-chip BrainScaleS-2 system. The ability to emulate and train networks larger than the substrate provides a pathway for accurate performance evaluation in planned or scaled systems, ultimately advancing the development and understanding of large-scale models and neuromorphic computing architectures.

## 1 Introduction

For traditional deep learning algorithms, whether simulated on conventional hardware or accelerated using GPUs and specialized hardware, the seamless integration of machine learning frameworks such as PyTorch (Paszke et al., 2019) and TensorFlow (Abadi et al., 2015) has simplified modeling and accelerated research. Recent years have seen a parallel evolution in the field of spiking neural networks (SNNs), where specialized modeling interfaces (Pehle and Pedersen, 2021; Manna et al., 2023) have begun to play a key role in streamlining the model development process. While the creation of a scaffold for building software support within machine learning libraries for general-purpose processing units is well established (Facebook Inc., 2021; Lattner et al., 2021), it is still an open research topic in the context of custom digital neuromorphic hardware (Shrestha et al., 2022), and even more so for the time-continuous nature of many analog neuromorphic systems, where the path to seamless integration is considerably more intricate.

In this work, we address typical model size limitations imposed by small substrates such as the BrainScaleS-2 (BSS-2) accelerated mixed-signal neuromorphic system (Pehle et al., 2022), which is currently only deployed in its single-chip variant. Initially, the BSS-2 architecture has been designed as a research vehicle for computational neuroscience, offering specialized features tailored to address the intricacies of neural dynamics and plasticity. The inclusion of multi-compartmental neurons, complex synapse dynamics, adaptive exponential integrate-and-fire (AdEx) compartment dynamics (Brette and Gerstner, 2005; Billaudelle et al., 2022), as well as short-term and long-term plasticity, positions BSS-2 as a versatile platform for exploring diverse neural phenomena. Beyond computational neuroscience, BSS-2 also extends its reach into machine-learning-inspired applications, where functional modeling often draws inspiration from machine learning.

Deep neural networks (DNNs) are often significantly larger than neuromorphic ASICs. While small-scale multi-chip system prototypes using an EXTOLL-based FPGA-mediated interconnect have been demonstrated (Thommes et al., 2022; Thommes, 2023), production BSS-2 system resources operate in single-chip configurations. However, networks with limited fan-in requirements that either comprise a pure feed-forward topology or sufficiently local recurrence allow for the partitioning into subnetworks that individually fit onto single ASICs. In general, partitioning introduces sequence points where emulation can be paused while the data flow still determines the execution order, i.e., subnetwork partitions of early layers are emulated before later layers, but the execution order within a layer is arbitrary. This therefore enables the sequential evaluation of networks larger than the existing neuromorphic substrate without having to resort to software simulation. Especially with regard to the typical costs and time required for hardware development, this enables early analysis and thus optimization of future hardware substrates. The reuse of “computational units” (neurons, synapses, routing, and other resources) is analogous to the way conventional von-Neumann architectures utilize computational resources and can be understood as a form of virtualization of the neuromorphic substrate. This departs from traditional neuromorphic systems, which allocate dedicated resources for each component of spiking neural networks. Recent work (Mysore et al., 2022) laid out a partitioning method for mapping large-scale neural network models onto neuromorphic hardware. Along these lines, for hardware supporting non-time-continuous operation, Song et al. (2020) describes a complete workflow from model specification to hardware execution. Previous work by the authors provided similar functionality for the activation-based —i.e. non-spiking— operation mode of BSS-2 (Spilger et al., 2020).

The BSS-2 software stack aims to provide a user-friendly modeling API that abstracts away from hardware-specific intricacies (Müller et al., 2022). Over the course of its development, machine learning inspired training approaches have become increasingly popular. However, until recently, our modeling efforts were mostly limited to the size constraints of single BSS-2 ASICs. In this work, we focus on providing a framework for integrating such partitioning methods more generally, particularly for large-scale SNNs, into the BSS-2 software stack. The method not only applies to single-chip substrates, but generalizes also to larger substrates by concurrently placing multiple partitions.

In this work, extend the capabilities of the BSS-2 platform to emulate larger-than-substrate-sized networks efficiently and seamlessly, thereby advancing the overarching goal to automate the process of making BSS-2 amenable for large-scale network. For this, we focus on scenarios, such as feed-forward networks or those with sufficiently small recurrent subnetworks, where hardware reuse becomes a practical proposition. Our new software feature introduces the manual partitioning into subnetworks for implicit sequential hardware execution, effectively abstracting away all hardware-related data flow of partitioned hardware runs from the top-level machine learning framework. We discuss our approach in the context of commonly used datasets and network topologies. Finally, we demonstrate the training and emulation of larger, multi-partition networks on single-chip BSS-2 substrates using the MNIST (LeCun et al., 1998b) dataset of handwritten digits and the EuroSAT (Helber et al., 2017) dataset for land use and land cover classification. The latter is of particular relevance for future applications in space (Izzo et al., 2022), as energy-efficient compute infrastructure such as neuromorphic hardware represents a promising candidate for neural solutions onboard spacecraft—especially miniaturized ones like CubeSats. We present the first results on BSS-2 for training functional networks larger than the hardware substrate.

## 2 Methods

**Figure 1:**
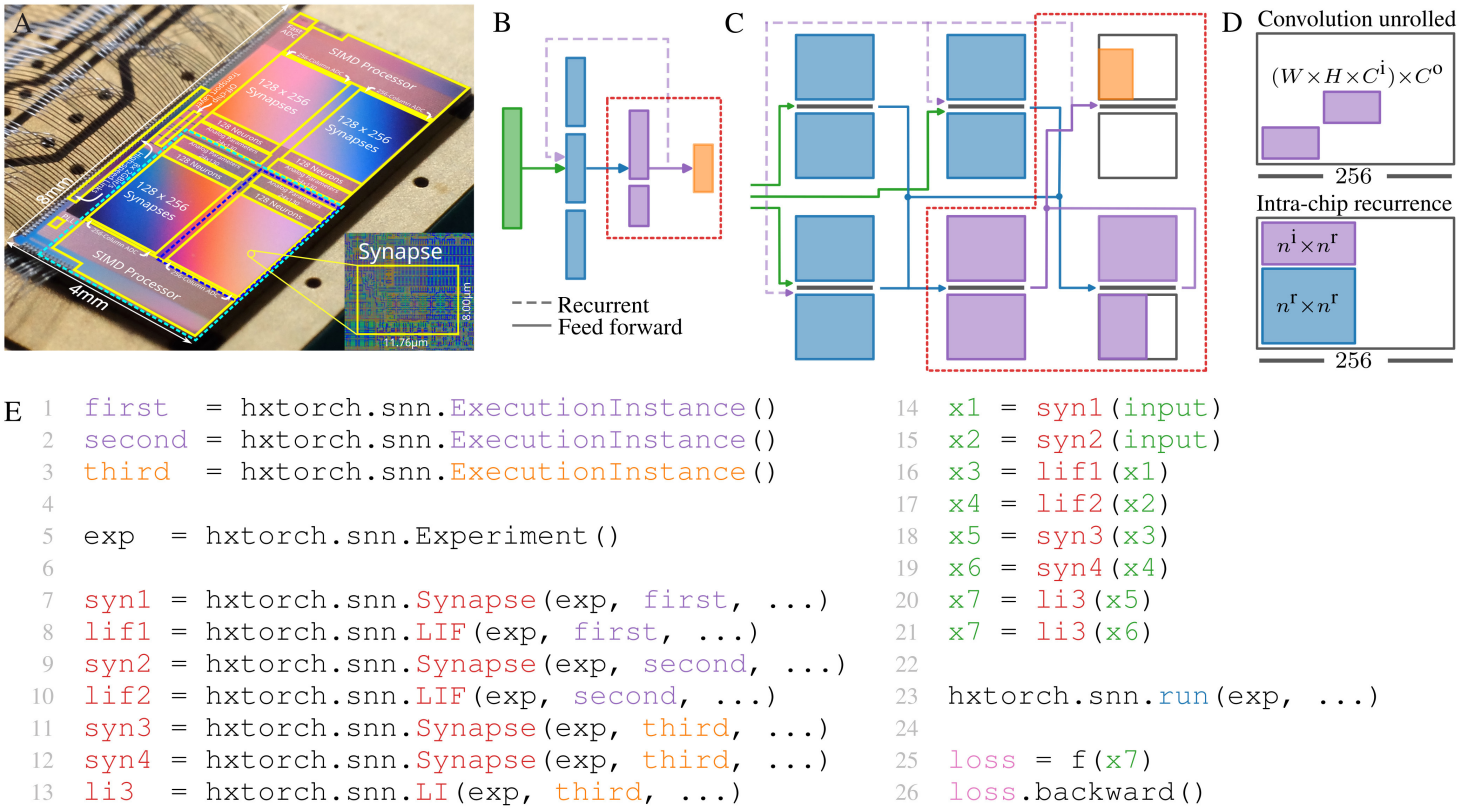
**(A)** A photo of the BSS-2 chip with its schematic overlaid on top. **(B)** A larger-scale network, exceeding the size of a single BSS-2 substrate. To emulate the full network, it can be partitioned into smaller subnetworks and executed concurrently on a multi-chip setup as displayed in **(C)** or all subnetworks are emulated sequentially by reusing the same chip resource. The concept of sequential execution also applies to networks that exceed scaled multi-chip system in size where the scaled system then becomes the largest sequentially allocatable entity. Dashed lines correspond to recurrent dependencies. **(D)** Upper: On BSS-2 convolutions need to be unrolled spatially thereby demanding excessive hardware resources and partitioning. Here, *W* and *H* corresponds to the width and height of the kernel, *C*^i^ and *C*^o^ are the number of input resp. output feature planes. Lower: For sequential network emulation, recurrent dependencies need to fit on a single substrate which reduces external fan-in. However, this limitation does not apply for concurrent network emulation. **(E)** Software API of explicitly partitioned network indicated by the dotted red line in **(B, C)**. ExecutionInstances are the software representation of a network topology and parameterization, which can be executed concurrently on the substrate, e.g., here one chip. For execution on hardware, first, the complete network topology is created. Execution is separated from the topology description by yielding *promise*s to future result data in x{1 − 7}. Only after execution on the hardware via run() are they filled with data and the loss can be calculated. Annotated backpropagation functions can then be used for gradient estimation.

In this work, the latest BSS-2 ASIC (Pehle et al., 2022) is used as a mixed-signal neuromorphic substrate, depicted in Figure 1A. It features 512 (single-compartment) neurons implementing the AdEx neuron equation in analog circuits (see Pehle et al., 2022 for details). The AdEx circuits can be configured to implement the leaky-integrate and fire (LIF) neuron dynamics used in this work, see Equations 1, 2 used in this work,


(1) $$\begin{array}{l} \tau_{\text{m}}\dot{v}=\left(v-E_{\text{l}}\right)+\frac{1}{g_{\text{l}}}I, \end{array}$$

with *v* being the membrane potential, τ_m_ the membrane time constant, *g*_l_ the leak conductance, and *E*_l_ the leak potential. The neurons support both conductance- and current-based synaptic input, here we use the latter. There, each neuron can receive input events at times $t_{i}^{\text{s}}$ from 256 pre-synaptic neurons {*i*} via synapses with weight *w*_*i*_, resulting in the synaptic input current *I*,


(2) $$\begin{array}{l} I\left(t\right)=\underset{\left\{t_{i}^{\text{s}}\right\}}{\sum }w_{i}\Theta \left(t-t_{i}^{\text{s}}\right)\exp-\frac{t-t_{i}^{\text{s}}}{\tau_{\text{s}}}, \end{array}$$

where τ_s_ is the synaptic time constant. If the membrane potential exceeds the neuron's threshold ϑ, the neuron emits a spike event and *v* is reset to the reset potential *E*_r_. Using on-chip routing, the maximum on-chip fan-out of a single neuron on BSS-2 is 2 × 32 × 2 × 256 = 32, 768 synapses.^1^ Each neuron supports a fan-in of 256, however, the fan-in can be increased by shortening multiple neuron circuits (see below). Events are propagated via digital signals, while the post-synaptic neuron dynamics evolve in the analog domain. Using the current default FPGA-ASIC link speed, the maximum sustained bandwidth is 250 MHz for both input and output events. Therefore, SNNs on BSS-2 are *emulated* time-continuously on an physical computing substrate and in real-time—in contrast to numerical *simulation*, the experiment in general cannot be paused. Hence, the network size which can be concurrently (and interdependently) emulated is limited by the number of neuron and synapse circuits, and other resources. However, concurrent placement and emulation is only required for tightly-coupled recurrent network subgraphs, while feed-forward network subgraphs can be partitioned and run in parts. Figure 1C sketches the partitioning of the feed-forward network in Figure 1B. Using a multi-chip substrate, the network can be emulated in continuous time. If there are no recurrent inter-chip dependencies (omitting dotted line in Figure 1B), the inter-chip communication does not need to happen in real time, and can be buffered. In that case, the whole network can also be emulated sequentially by reusing a single chip. Since convolutions need to be spatially unrolled on BSS-2 (see Figure 1D), spiking convolutional networks on BSS-2 will benefit from the presented feature.

Splitting networks into multiple partitions and emulating them sequentially requires the events in-between executions to be recorded and played back in dependent executions. This increases the required communication of events from and to the system compared to direct forwarding of events within the hardware. However, for typical machine-learning-inspired training the readout of events from hidden layers is required in any case.

Partitioning projections does not necessarily decrease the fan-in for the post-synaptic layer, since neuron dynamics are not linear. Thus, we take advantage of the hardware's ability to combine neuron circuits, resulting in an increased fan-in capability of “256·#neuron circuits per neuron,” up to the complete chip, i.e., 256 × 512= 131,072 unsigned weights. We use two 6 bit-weight hardware synapses to represent a signed weight, therefore the maximum number of signed input weights is 65,536. Consequently, this decreases the number of “logical” neurons available per single execution by #neurons = 512#neuron circuits per neuron, possibly increasing the number of required partitions.

We base our work on the existing BSS-2 software stack, which provides multiple abstraction layers, see Müller et al. (2022) for details. Specifically, we integrated partitioned execution functionality into the layer that represents experiments as a signal flow graph. Even before this support was added, the signal flow graph had an understanding of data input and output operations, so the addition of temporary readout and data reinsertion functionality was a natural extension. To take advantage of developments in the machine learning community, the user-facing hxtorch API (Spilger et al., 2023) is based on PyTorch data structures and integrates with its auto-differentiation functionality.

### 2.1 Training

The MNIST and EuroSAT models are trained using well-established surrogate gradient-based learning methods (Neftci et al., 2019). Class scores are optimized by minimizing the cross-entropy loss, using the Adam optimizer (Kingma and Ba, 2014) with (surrogate) gradients obtained by the backpropagation through time (BPTT) algorithm. To approximate the networks' gradients on BSS-2, we apply the hardware-in-the-loop (ITL) training procedure (Schmitt et al., 2017) and record and read out the network observables, i.e., membrane voltages and spikes. These observables are mapped to PyTorch tensor data structures with a fixed time grid with resolution δ*t*. For this, we calculate the factor which scales the membrane dynamics on BSS-2 to the corresponding dynamics in software, that are idealized for gradient estimation. Synapse and neuron dynamics are numerically integrated on this time lattice in the case of simulated (sub-)networks. Each part of the network is run, or simulated respectively, for *T* = 30 µsg in the case of MNIST and 64 μs for the EuroSAT task per image. The measured/simulated membrane traces *v*_*k*_ in the readout layer are converted into scores *s*_*k*_ via a max-over-time decoding, $s_{k}=\underset{t}{\max}\left(v_{k}\left(t\right)\right)$ (Cramer et al., 2022) for MNIST, or by taking the last observed membrane value *s*_*k*_ = *v*_*k*_(*T*) for the EuroSAT dataset. The partitioning of the considered SNNs is explained in Section 3.

### 2.2 MNIST

The MNIST (LeCun et al., 1998b) dataset contains 70 000 28 × 28 gray scale images of handwritten digits that are categorized into 10 classes (0 to 9). 60,000 images are meant for training purposes, the rest for testing the model. We consider a fully connected feed-forward network with 256 LIF units in the hidden layer and 10 leaky integrators (LIs) in the readout layer. A time-to-first spike (TTFS) encoding scheme, described in Section 3.2.1, transfers the images from a pixel-value representation to spike events. The dataset is augmented by using random rotations up to 25° which are applied with a probability of 50%, additionally we normalize images. For improved generalization we also use dropout with a probability of 15% in the hidden layer, resulting in some of the hidden spikes not being injected into the readout layer during training. To keep the network's dynamics and parameters within the system capabilities, we use regularization terms for the firing rate in the hidden layer which might exceed the system's bandwidth, the readout membrane traces which might saturate due to the limited range of the columnar ADC (CADC) and the weights which are also limited in range on hardware. The training process spans 100 epochs during which the learning rate and firing rate regularization constant decrease exponentially. At the end of the training, the model's performance is evaluated with the test set. The final performance is the averaged over different pseudorandom number generator (PRNG) seeds. A summary of the used training and model parameters is given in Supplementary Table 1.

### 2.3 EuroSAT

The EuroSAT dataset consists of 27.000 64 × 64 × 3 RGB^2^ images of the Earth's surface taken by the satellite mission Sentinel-2, categorized into 10 classes. We split the dataset in training, validation, and test set by ratios 0.7, 0.1, and 0.2. For regularization, random flips are applied to the training images. For its classification, we consider a network with two hidden LIF layers consisting of 484 and 128 units, and one LI readout layer to infer decisions. For spike encoding of the input images we use a TTFS encoding, described by Equation 4. In addition to the training procedure outlined in Section 2.1, we halve the learning rate after the epochs {10, 20, …, 60}. Training is performed for a maximum of 500 epochs in simulations, or 100 on BSS-2. If there is no improvements on the validation accuracy for 25 epochs in simulation or 15 epochs on BSS-2, the training is stopped. We save the best performing model on the validation set and use it for later evaluation on the test set. A summary of all model and training parameters is given in Supplementary Table 2.

## 3 Results

In this section we describe our implementation, which introduces software support for model partitioning and sequential execution on BSS-2. We demonstrate its use on the MNIST and EuroSAT datasets.

### 3.1 Software

While the user of a machine learning framework does not need to know the partitioning, this information is required in the intermediate representation used for scheduling execution on the hardware. In the high-level experiment description, networks are comprised of populations of neurons and projections of synapses. We use a signal-flow graph to represent multiple executions and their data-flow dependencies. This representation can be used to represent partitioned networks. To this end, network entities are annotated with information regarding their associated execution (ExecutionInstance in Figure 1E). The inter-execution projection represents the forwarding of events from one execution to another. It receives recorded events from the source execution and injects these events into the target execution. The host computer is used for the translation of the events, which allows for the complete decoupling of event routing constraints between executions.

In our machine learning frontend hxtorch.snn, each layer is assigned to a specific execution via a parameter upon construction. The inter-execution dependencies are then automatically extracted from the network topology. This enables explicit (manual) partitioning as well as employing user-defined partitioning algorithms, which can also be used for mixed hardware-emulated and software-simulated networks, see Section 3.2.2. Figure 1E shows a frontend API example.

It is anticipated that the utilization of multiple partially sequential executions and the increased required data transfer when using multiple partitions in contrast to executing a network in a single hardware run will result in a reduction in runtime performance. The hardware runtime scales linearly with the depth of the partitioned network, since these executions are required to be run sequentially due to inter-partition data dependencies. Partitions without data dependencies, e.g., multiple partitions of the same layer, can be executed concurrently. The choice of whether to execute the partitions concurrently or sequentially depends on the available hardware resources. Therefore, runtime additionally scales linearly with the ratio of concurrently executable partitions to available hardware. When using partitioning, all events between partitions are recorded and translated on the host computer. In contrast, networks executed in a single non-partitioned hardware run only require complete event recording during training, as only the data from the last layer is typically of interest during inference. In addition, event recording and translation overhead is expected to impair runtime performance in comparison to non-partitioned experiments. A dedicated inter-execution memory buffer in some field-programmable gate array (FPGA)-managed dynamic random-access memory (DRAM) could at least eliminate the software overhead at the cost of additional FPGA development effort to support additional translation and playback of recorded data. Table 1 shows wall-clock runtime measurements of the MNIST experiment, cf. Section 3.2.1, broken down to evaluate the performance impairment attributed to partitioned execution. Here, membrane potential recording dominates the hardware runtime, which is potentiated by the linear scaling with the number of partitions. Event recording and playback via the host computer on the other hand is insignificant.

**Table 1:** Wall-clock duration measurements (top) and user-requested minimal realtime runtimes (bottom) for the model classifying MNIST, cf. Section 3.2.1, for a single batched execution of 100 images with 30 μs experiment runtime each.

**Experiment step**	**Duration**	**Data**
Host computer compilation & post-processing	692 ms	
Event encoding	0.3 ms	721 spikes
Event decoding	0.7 ms	909 spikes
Membrane recording decoding	100 ms	8,445 samples
Hardware experiment total	248 ms	
ML front end data handling, backward pass	810 ms	
Total	1,800 ms	
Partitioned hardware runtime (5 partitions)	40 ms	
Realtime hardware runtime (per partition)	3 ms	
Inter-batch-entry hardware wait (per partition)	5 ms	

In-between batch entries, for relaxing the analog neuron dynamics, a wait period of 50 μs is added additionally, resulting in a minimal hardware runtime of 8 ms. Since the model is partitioned into five sequential executions, this minimal runtime is scaled linearly to 40 ms. The difference to the measured total hardware runtime of 248 ms is attributed predominantly to recording the neuron's membrane potential during the experiment, which also additionally yields 100 ms of host computer runtime. Event decoding is required for training, only event encoding of 0.3 ms is attributed to partitioning and sequential execution, which is deemed insignificant. While this results in an overall overhead of a factor of 600 (or 225 when accounting for the relaxation/wait time) between the minimal experiment runtime and the training wall-clock runtime using partitioning, we expect the same experiment to run a factor of five faster (same as number of partitions) on a sufficiently large substrate that allows training without partitioned sequential execution.

**Figure 2:**
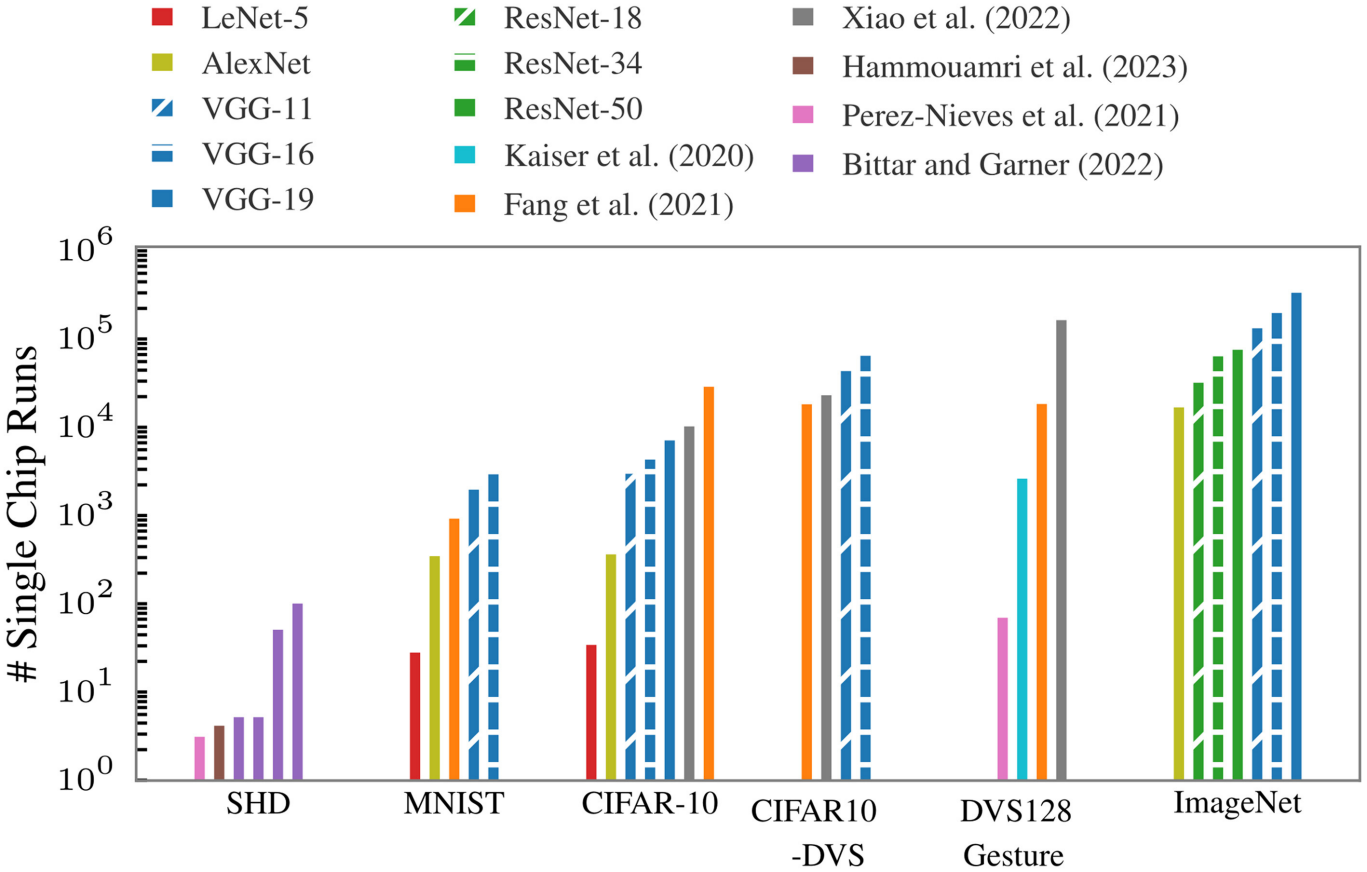
Projected single-chip runs needed for different networks topologies used for different datasets. The resources required for convolutional networks depend on the size of the inputs. We assume that the convolutional layers are unrolled spatially. For Bittar and Garner (2022), the first two bars correspond to networks with 128 hidden neurons, the last two have 1,024 neurons. The experiments indicated by the first and third bar use feed-forward networks, the ones represented by the second and forth bars use networks with recurrent connections.

In Figure 2, we project the single-chip runs required for our presented approach for various network topologies, i.e., LeNet (LeCun et al., 1998a), VGG (Simonyan and Zisserman, 2015), and ResNet (He et al., 2016), including those used in the referenced publications. The considered datasets are Spiking Heidelberg Digits (SHD), MNIST (LeCun et al., 1998b), CIFAR-10 (Krizhevsky, 2009), its event-based version CIFAR10-DVS (Cheng et al., 2020), DVS128 Gesture (Amir et al., 2017), and ImageNet (Deng et al., 2009). These network topologies and datasets represent a broad spectrum of different input sizes and required topological complexities. We assume all convolutional layers need to be spatially unrolled, with pooling operations and normalization, e.g., batch normalization, either integrated into the weight layers or processed in the event domain. Furthermore, the presented numbers are subject to optimization through advanced mapping algorithms and may vary based on the implementation of certain operations (e.g., pooling or residual connections) in future large-scale neuromorphic hardware. The significant number of single-chip runs required to emulate common model topologies for real-world problems underscores the critical importance of our approach to partitioning and sequential model execution—even as large-scale multi-chip hardware becomes available in the future.

### 3.2 Examples

We exemplify our support for partitioning using SNN models with topologies that otherwise would not be emulatable on a single-chip BSS-2 system.

#### 3.2.1 MNIST

**Figure 3:**
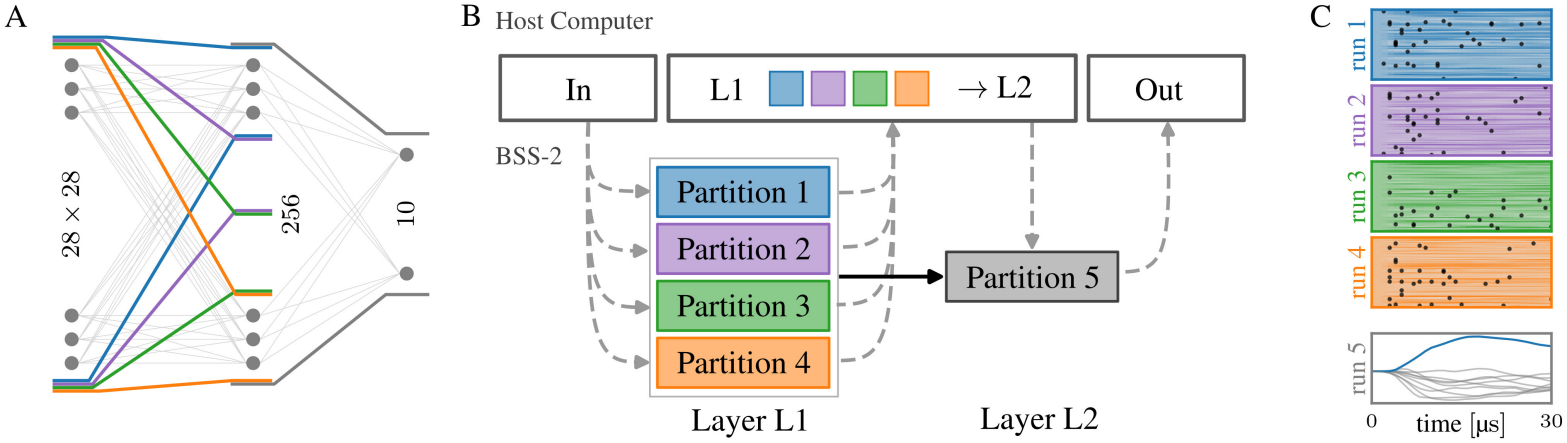
**(A)** Schematic network topology for a network of 28 × 28 → 256 → 10 neurons. Partitions that can be run consecutively on hardware are marked. The four partitions in the first layer are interchangeable. **(B)** Data flow of the model from **(A)** using five partitions, where the additional need to record and play back events to/from the host computer in-between layers is visualized by dashed lines. **(C)** Measured spikes and membrane potentials of each hardware run. To run the fifth partition, the spikes from the first four partitions need to be known. On a multi-chip setup with at least five chips, all parts could be run in parallel.

Executing the network described in Section 2.2 with the single-chip BSS-2 system is only possible after partitioning it into five parts as the 28 × 28 inputs require multiple neuron circuits to be connected, see Figure 3A. Specifically, the 784 pixels are mapped to the same number of signed weights per neuron, requiring two hardware synapses each, thereby requiring eight^3^ combined neuron circuits. By partitioning the hidden layer of 256 units into four parts, the 64 units per partition comply with the BSS-2 substrate (64 × 8 = 512, the number of neuron circuits on the chip) so that each of the parts can be executed in one run. For each run, the input events need to be provided, as indicated by the dashed lines in Figure 3B, which showcases a schematic view of the network and the necessary partitions for execution on BSS-2. Once the spike events have been read out from the four parts of the hidden layer they are reassembled in software which is required to emulate the readout layer. The observed spikes on BSS-2 for each partition and the membrane traces of the output layer are shown in Figure 3C.

The particular TTFS encoding used here assigns spike times $t_{i}^{s}$ to pixel values *x*_*i*_ in a linear manner,


(3) $$\begin{array}{l} x_{i}\to t_{i}^{s}=\left(T-\left\lfloor \frac{T}{\delta t}\cdot \frac{x_{i}-x_{\text{min}}}{x_{\text{max}}-x_{\text{min}}}\right\rceil \cdot \delta t\right) \end{array}$$

where *T* is the sequence length per image, that together with the time interval δ*t* determines the encoding resolution. The mixed flooring and ceiling brackets indicate rounding to the next integer and *x*_min/max_ are the minimum/maximum pixel values of the dataset. All previous publications reporting on this benchmark on BSS-2 used a scaled-down image size of 16 × 16 to reduce input dimensionality in order to fit the whole network on a single chip instance, compare Table 2. Our model is the first implementation using the full resolution of 28 × 28 on BSS-2—and a slightly larger hidden layer (256 compared to 246 before; see Figure 3A)—and reaches 97.9(1)% using similar training methods. Although the slight improvement in classification performance does not indicate the necessity for the development of means to run larger-scale models, it represents an important milestone in the validation of our implementation and hardware operation against previous results.

**Table 2:** MNIST experiments on BSS-2.

**Publication**	**Input size**	**Test accuracy [%]**
Göltz et al. (2021)	16 × 16	96.9 ± 0.1
Cramer et al. (2022)	16 × 16	97.6 ± 0.1
This work	28 × 28	**97.9 ± 0.1**

Bold indicates the best result.

#### 3.2.2 EuroSAT

**Figure 4:**
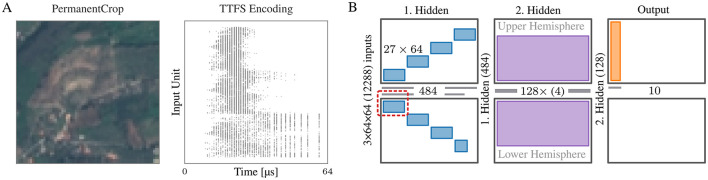
**(A)** (left) Example image of the EuroSAT dataset. (middle) The image TTFS encoded. **(B)** Partitioning and placement of the network used to classify the EuroSAT dataset. The basic synapse and neuron layout of the BSS-2 ASIC is shown in each column: in the center, two rows of neuron circuits are located; each neuron row is fed from the adjacent synapse array (top/bottom rectangles). Neuron circuits can be combined to form larger logical neurons, supporting larger fan-in. On the left of each hardware instance, the source and size of the fan-in are indicated. Each neuron in the first hidden layer has a receptive field of 3 × 3 × 3 and can be mapped to one BSS-2 instance. To reduce the number of input spikes, we run it in multiple parts (indicated by the red box). The neurons in the second layer consist of four connected neuron circuits. This layer, as well as the readout layer, is executed in a single run each.

We trained the model described in Section 2.3 to classify the EuroSAT dataset (Helber et al., 2017). Its partitioning and placement on BSS-2 is visualized in Figure 4B. Instead of densely projecting the large input space onto the first hidden layer, each neuron in the layer has a small receptive field of 3 × 3 × 3 pixels. The receptive fields are moved over the spatial coordinates (height and width) of the image with stride 3, resulting in each neuron receiving a unique set of input pixels. For the BSS-2 system this encoding is particularly convenient since it makes uses of the system's intrinsic support for placing sparse connections. With the given size of the receptive field, the first hidden layer has a size of 484 neurons with 27 inputs each. Each synapse row on BSS-2 can distinguish 64 event labels, hence, we uniquely address a maximum of 64 neurons through the same row. This allows to map the sparse projection in blocks of 27 × 64 “signed” hardware synapses onto BSS-2 and thus run the whole first layer at once. The large input space in conjunction with the used TTFS encoding scheme still results in a fair amount of spikes, hence, means for reducing the number of input events are applied—also by partitioning of the first hidden layer, thereby reducing the number of input neurons required per execution (see red box in Figure 4B). We execute this layer in 8 parts, resulting in 10 runs needed to emulate the whole network. The remaining projections between layers have all-to-all connectivity. The second hidden layer of size 128, can be emulated within one run by connecting four neuron circuits on BSS-2 to form one neuron in order to support a fan-in of 484 from the previous layer. The readout layer is implemented with single-circuit neurons.

To avoid the on-chip spike event rate to exceed the system's bandwidth, we use an TTFS input encoding scheme, see Figure 4A. Each pixel value *x*_*i*_ ∈ [0, 1] is interpreted as a constant current onto a LIF neuron with an infinite refractory period, i.e., the neuron can only spike once at $t_{i}^{\text{s}}$ (cf. Cramer et al., 2022). This yields an early spike time for stronger pixel intensities and no input spike if the pixel value is too small. We add a bias value *x*_min_ to *x*_*i*_ to bias the inputs toward early spiking. The spike times $t_{i}^{\text{s}}$ are numerically computed according to


(4) $$\begin{array}{l} x_{i}\to t_{i}^{\text{s}}=t{|}_{v_{i}\left(t\right)=\vartheta_{\text{en}}}\text{ with }\dot{v_{i}}\left(t\right)=-\frac{1}{\tau_{\text{en}}}v_{i}\left(t\right)+x_{i}+x_{\text{min}}, \end{array}$$

with *v*_*i*_ being a membrane state, and ϑ_en_ a threshold. See Figure 4A for an example. Using this encoding, we achieve an average spike count per time bin of 162 (averaged over training set and time bins) and the maximum average spike count encountered in a bin (averaged over training set) to 527.

The BSS-2 FPGA only processes two spikes per clock cycle, i.e. simultaneous sends might get delayed. If the maximum bandwidth is exceeded for longer time spans, spikes are dropped. To minimize simultaneous events, we compute the spike times at FPGA resolution. However, since the dataset is constituted of only 252 unique pixel values only the same number of unique spike times will occur. In the forward direction, we therefore jitter the pixel images by adding Gaussian noise, $x_{i}+N$(μ = 0, σ_in_). For gradient optimization we assume the same resolution δ*t* as in the simulations. All parameters are summarized in Supplementary Table 2.

**Figure 5:**
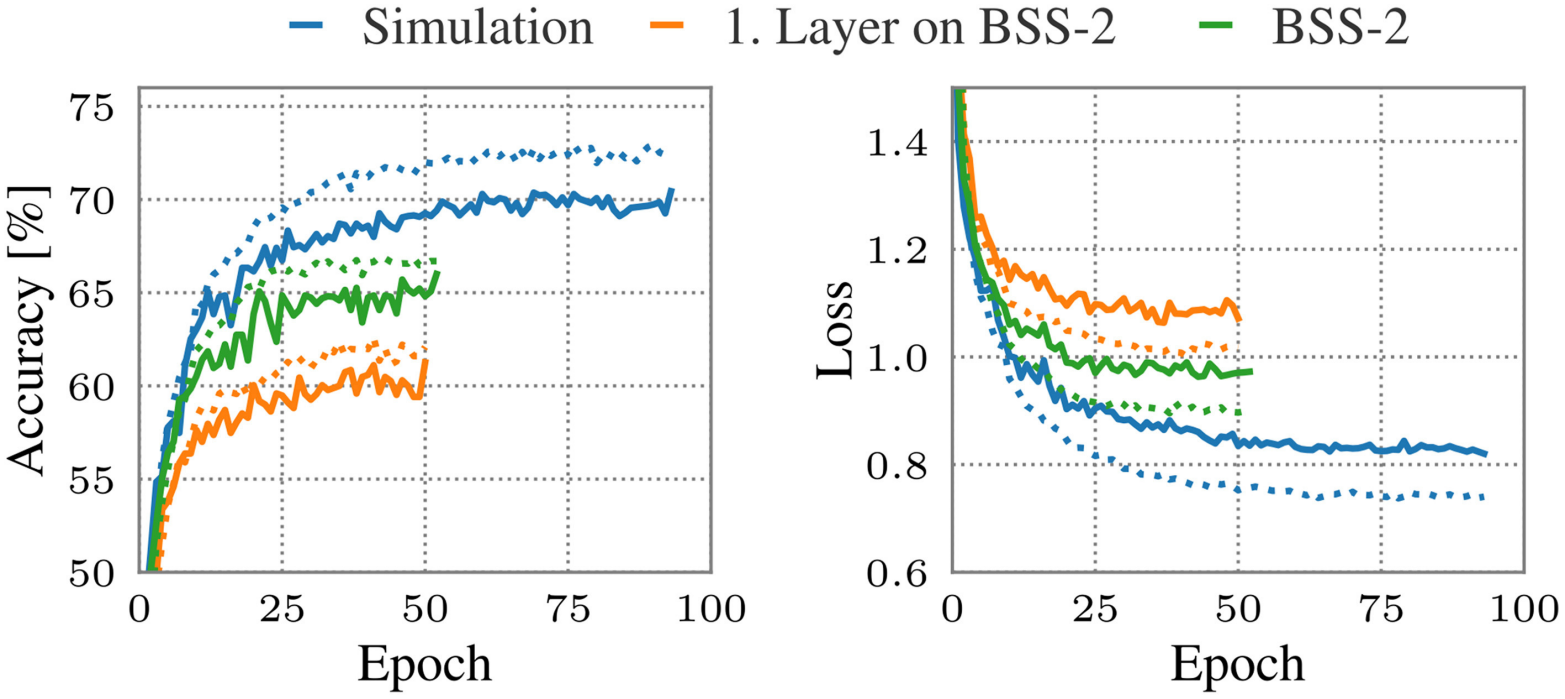
Accuracy **(left)** and loss **(right)** of the model on the EuroSAT dataset in simulation and/or on BSS-2. The dotted lines correspond to the training set, the solid to the validation set. Blue corresponds to a fully simulated network, green to the whole SNN partitioned emulated on BSS-2, and orange to mixed simulation/BSS-2 execution with only the first layer being emulated on BSS-2.

In Figure 5 we show the training (dotted) and validation (solid) accuracy and loss of our model on the EuroSAT dataset. We achieve a test accuracy of 69.6% (blue) in a software-only training. When emulating the whole model on BSS-2 (green) the test accuracy is 60.65%. We showcase an example of mixed numerical simulation/BSS-2 emulation where only the first hidden layer is run on BSS-2 (orange). A penalty of approximately 9% is observed on BSS-2, with approximately 50% of this value attributable to the first hidden layer, as indicated by the mixed simulation/BSS-2 experiment. This emphasizes the importance of support for mixed execution to investigate and improve the performance of future models and systems.

## 4 Discussion

This paper emphasizes the role of software in enabling the partitioned emulation of large-scale SNNs on the BSS-2 neuromorphic substrate. While manual partitioning of suitable SNN topologies has always been a viable approach, the integration of software support into the BSS-2 software stack enables researchers to shift their focus from system handling to modeling. The present work is concerned with enabling the expression of manually partitioned networks, with the aim of enabling a rapid adoption by modelers. Future developments will aim to provide automated algorithms for partitioning, thereby relieving users of this task and enabling the creation of more complex partitioned network topologies.

We demonstrated partitioned emulation on SNN models classifying the MNIST and EuroSAT datasets, which require the use of many single BSS-2 chip instances. While the training processes used surrogate gradient-based learning methods (Neftci et al., 2019), an event-driven training approach, e.g., using the EventProp algorithm (Wunderlich and Pehle, 2021) and the event-driven BSS-2 modeling API jaxsnn (Müller et al., 2024), could provide further efficiency gains by exploiting sparsity in observables, thereby minimizing data transfers between host and neuromorphic hardware, as well as in numerical computations.

To validate our implementation, we used the MNIST dataset, as there are several publications using single-chip BSS-2 systems. Our model performs slightly better on 28 × 28 image resolution than the smaller models on 16 × 16 images, achieving 97.9(1) % test accuracy. For further details, please see Section 3.2.1. This represents the best performance on MNIST recorded on BSS-2 to date. We acknowledge that this improvement may also be partially attributable to a more efficient input encoding and training setup. This is the first time the full-scale benchmark has been run on BSS-2. The capacity to benchmark systems without the necessity for extensive pre-processing and downscaling ensures fair comparison to other systems, thereby underscoring the importance of facilitated partitioned emulation of SNNs on small-scale systems.

For the larger EuroSAT task, we present the first results obtained on BSS-2. We showcase the emulation of the largest SNN to date on BSS-2 through the partitioning into subnetworks, each of which is executable on the available hardware substrate. The sparse input projection enables us to map a 12288-dimensional input space to the hardware. Due to connectivity sparsity, the first hidden layer is emulated in eight parts, resulting in ten partitions for the whole network. In the future, sufficiently large multi-chip systems will be capable of emulating all partitions concurrently. The sequential execution of the model on BSS-2 resulted in a test accuracy of 60.65 %, thus supporting our presented approach for large-scale model emulation. The performance gap to the numeric simulation is assumed to be not intrinsic to the analog nature of the system. Potential causes for the observed performance degradation on BSS-2 include suboptimal hardware operation points and training setup, in addition to spike loss in the input layer due to bandwidth constraints. We are optimistic to resolve the latter by stretching the experiment in time to minimize the number of simultaneous events and by increasing the number of partitions of the first hidden layer. Our support for emulating only parts of the network on BSS-2 and numerically simulating the remaining parts is a crucial feature for identifying hardware-specific intricacies and debugging the model's performance, e.g., by identifying which dynamics of the SNN are emulated at a suboptimal hardware operation point.

While partitioned emulation is typically superlinearly slower than on a sufficiently large substrate, the ability to explore larger networks is valuable, especially when considering typical hardware development cycle times and costs. We have shown this superlinearity for the MNIST experiment, where the inter-execution data transfer via the host however is insignificant, leaving the linear scaling to the preparation, execution and post-processing of the sequential executions.

Due to the mixed-signal nature of the BSS-2 architecture—and many other neuromorphic systems (Thakur et al., 2018)—the partitioning of SNNs does not affect the emulation fidelity compared to a system with network-matching system size: spikes are events in time that can be reliably recorded (within the constraints of the system's I/O bandwidth) and played back at later points in time, thereby providing deterministic communication between subnetworks. The ability to facilitate answering questions about the desired model and hardware system size with the confidence of a realistic emulation is a key outcome of this work. This not only addresses the immediate need to understand the behavior of larger networks on existing hardware, but also provides valuable insight into the feasibility and performance expectations for future, more expansive —and expensive—neuromorphic systems.

## Data Availability

Publicly available datasets were analyzed in this study. The EuroSAT dataset can be found here: https://github.com/phelber/EuroSAT. Researchers can use the EBRAINS research infrastructure to access BrainScaleS-2 systems: https://www.ebrains.eu/nmc. An MNIST example can be found in the BrainScaleS-2 tutorial collection: https://electronicvisions.github.io/documentation-brainscales2/latest/brainscales2-demos.

## Footnotes

^1^Using off-chip routing, the maximum fan-out is typically limited by finite routing tables and link bandwidths.

^2^We only consider the RGB bands out of the 13 provided spectral bands.

^3^Actually seven, but to simplify the mapping onto BSS-2, eight circuits are used.
