# Supplementary material for: Scalable network emulation on analog neuromorphic hardware
Source: Front Neurosci. 2025 Feb 5;18:1523331. doi: 10.3389/fnins.2024.1523331 (PMC11835975; doi:10.3389/fnins.2024.1523331)
Supplement: Supplementary file 1 [file Data_Sheet_1.pdf]

SUPPLEMENTAL DATA

Table 1 and table 2 provide parameters for the MNIST and EuroSAT experiments.

Table 1. MNIST Experiment

**Training**	
Batch size	100
Learning rate	0.002
Epochs	100
Learning rate decay	0.985
Vertical/Horizontal flip	25 % probability
Dropout	0.15
Optimizer	Adam (default)
SuperSpike slope α	50
**Simulation & Gradient**	
δt	1 µs
Time steps T	30
Leakage potential	0
Reset potential	0
Threshold	1
Mem. time constant	6 µs
Syn. time constant	5.7 µs
Readout scale	3
**Regularization**	
Bursts	0.0025
Θ_{h}	0.0033
Θ_{o}	0.0033
v_{o}	0.00016
γ	0.985
**Encoder**	
x_{min}	0
x_{max}	1
**BSS-2 Operation Point**$^{\triangle}$	
`i_synin_gm`*	[800, 400]
`synapse_dac_bias`*	[850, 700]
`leak`	80
`reset`	80
`threshold`	120
`membrane_capacitance`	63
`refractory_time`	1 µs

Table 2. EuroSAT Experiment

**Training**	
Batch size	64
Learning rate	0.001
Max. epochs	25 / 15
Learning rate decay	0.5 every $10, \ldots, 60$
Vertical/Horizontal flip	50 % probability
Optimizer	Adam (default)
**Simulation & Gradient**	
δt	1 µs
Time steps T	64
Leakage potential	0
Reset potential	0
Syn. time const.*	$[10, 10, 10]$µs
Mem. time const.*	$[10, 10, 10]$µs
Thresholds ϑ*	$[1, 1, -]$
SuperSpike slope α*	$[10, 10, -]$
**Encoder**	
Threshold ϑ_{en}	0.32
Time constant τ_{en}	20 µs
x_{min}	0.1
σ_{in}	0.003
**BSS-2 Operation Point**$^{\triangle}$	
`i_synin_gm`*	[350, 350, 300]
`synapse_dac_bias`*	[1000, 1000, 600]
`leak`*	[100, 100, 120]
`reset`*	[100, 100, 120]
`threshold`*	[160, 160, 140]
`membrane_capacitance`	63
`refractory_time`*	[1, 1, 0.4]µs

$^{\triangle}$ Integer numbers are either digitally settable to a set of specific values (e.g., `membrane_capacitance`), digitally settable to a range of values (e.g., `i_synin_gm`), or calibrated and given in on-chip measured "ADC" units (e.g., `threshold`) as used by the calibration library `calix`.
* List index corresponds to layer index.
